# Consumers Turning to the Internet Pharmacy Market: Cross-Sectional Study on the Frequency and Attitudes of Hungarian Patients Purchasing Medications Online

**DOI:** 10.2196/11115

**Published:** 2018-08-22

**Authors:** András Fittler, Róbert György Vida, Mátyás Káplár, Lajos Botz

**Affiliations:** ^1^ Department of Pharmaceutics Faculty of Pharmacy University of Pécs Pécs Hungary; ^2^ Institute of Psychology Faculty of Humanities University of Pécs Pécs Hungary

**Keywords:** survey, internet pharmacy, online medications, Hungary, attitude

## Abstract

**Background:**

During the past two decades, the internet has become an accepted way to purchase products and services. Buying medications online are no exception. Besides its benefits, several patient safety risks are linked to the purchase of medicines outside the traditional supply chain. Although thousands of internet pharmacies are accessible on the web, the actual size of the market is unknown. Currently, there is limited data available on the use of internet pharmacies, the number, and attitude of people obtaining medications and other health products from the internet.

**Objective:**

This study aims to gather information on the frequency and attitudes of patients purchasing medications online in a nationally representative sample of outpatients. Attitudes towards main supply chain channels, perceived benefits, and disadvantages of influencing online medication purchase are evaluated.

**Methods:**

A cross-sectional explorative study using a personally administered survey was conducted in a representative sample of Hungarian outpatients in 2018.

**Results:**

A total of 1055 outpatients completed the survey (response rate 77.23%). The mean age was 45 years, and 456 (43.22%) reported having chronic health conditions. The majority (872/1055, 82.65%) of the respondents were aware that medications could be obtained online, but only 44 (4.17%) used the internet for previous medication purchases. Attitudes towards the different pharmaceutical supply chain retail channels showed significant differences (*P*<.001), respondents accepted retail pharmacy units as the most appropriate source of medications while rejected internet pharmacies. Respondents were asked to evaluate 9 statements regarding the potential benefits and disadvantages about the online medicine purchase, and based on the computed relative attitude rate there is a weak still significant tendency toward rejection (*P*<.001). Correspondence of demographic factors, internet usage behavior, and prospective online drug purchase attitude was evaluated. Respondents who use the internet more and purchase goods online will be more likely to buy medications online. Furthermore, youth and education will determine the medication purchase behavior.

**Conclusions:**

Many patients will purchase medications on the internet in the future. Currently, there is an increased risk of patients buying products from illegal sites because these dominate the global online pharmacy market. Consequently, improved patient-provider communication and promotion campaigns are needed to inform the public about the safe use of internet pharmacies, as these initiatives can directly prevent patient safety threats.

## Introduction

### The Internet Market of Pharmaceuticals

The internet has revolutionized and changed our lives, communication, and procurement practices and strategies [1]. As access to the internet increases, its use to seek health information is also expanding. Estimates worldwide show that approximately 4.5% of all internet searches are linked to health-related questions or information [2]. Population-based surveys found that 72% of the online population in the US and 71% of internet users in Europe, searched for health information at least once in the previous twelve months [3-5]. These tendencies are further extended by mobile device uses [6]. However, consumers turn to the internet today not only for retrieving health information, but also to self-diagnose and obtain various health services or products [7,8].

According to an early definition by Fung et al [9], an online pharmacy is an internet-based vendor (legal or illegal), which sells medicine and may operate as an independent internet-only site, an online branch of “brick-and-mortar” pharmacy, or sites representing a partnership among pharmacies. Briefly, an online pharmacy is a website offering to deliver, distribute, or dispense medication on the internet directly to consumers [10,11]. The growing market of online pharmacies is facilitated by the rapid expansion of the internet, the ever-increasing digital health, the shift towards self-diagnosing from the direct doctor-patient relations, consumer experience in online purchases, the ease of mail-order trade, and distance selling [12,13].

The internet’s supply of pharmaceuticals has developed in numerous ways and according to different models in each part of the world. This is due to diverse regulatory, economic, and cultural environments. In the US, the internet pharmacy market is mainly prescription based, while throughout Europe, this segment is forming according to a nonprescription based model [14]. Today, internet pharmacies can be accessed globally. Thus, the legislative and economic perspectives should be considered throughout every country, worldwide. Therefore, online pharmacies generate regulatory confusion as pharmaceuticals and health services “move” between jurisdictional boundaries. Hence, the countries of operation and delivery must be evaluated [15]. While the country of operation determines the licensing requirements and the quality assurance standards in support of the practice of internet selling of medications, mail-order must be performed in accordance with the latter. However, since many illegitimate websites are unwilling to indicate their actual location, one cannot be certain of the regulatory framework under which the internet pharmacy is operating [15]. It is further complicated by the fact that national authorities are typically powerless beyond their borders [16,17].

There are several patient safety risks linked to the online purchase of medicines outside the traditional supply chain, including counterfeit medications. The proportion of counterfeit medicine is estimated to be 10% worldwide [18] ranging from less than 1% in the developed countries [19] to over 30% in developing countries such as Africa, Asia, India, and Latin America [20,21].

Illegal actors primarily focus on the uncontrolled sale of prescription drugs outside the regulated drug supply system [22]. Their marketing strategy includes emphasizing the most commonly preferred benefits of online pharmacies (convenience, speed, discounts, privacy, not visiting the physician, bulk orders and discounts, bonus medicine as a gift) and retaining information regarding adverse effects, contraindications, and drug interactions [21]. Nearly every therapeutic category of drugs is available through the internet. Not only the performance and image-enhancing and “lifestyle drugs” [23,24], such as phosphodiesterase type 5 inhibitors [25-27] or anti-baldness products [28], but life-saving medicines (eg, from the World Health Organization Essential Medicines List), analgesics (nonsteroidal anti-inflammatory drugs, opioids) [29,30], psychiatric [31], obesity [32,33], and cardiologic drugs [18] can be purchased freely over the internet.

The primary characteristics of this illegal market segment consists in the trading of seemingly identical products in an uncontrolled environment, with no restrictions on the consumers (eg, people under 18 can also purchase medications via the internet) or on products (larger quantities can be purchased) from a large virtual supply [18,21,34,35]. During the past two decades, the internet has become an accepted way to purchase medications due to convenience, the potential to save money, and privacy. Early reports on the use of the internet for buying drugs indicate the practical reality of obtaining prescriptions or purchasing prescription drugs online is very small [36]. However, recent reports suggest that the use of internet pharmacies, the number of people obtaining medications, and other health products online is increasing [37].

### Aims

This study aims to gather information on the frequency and attitudes of patients purchasing medications online using a nationally representative sample of outpatients in the Southern Transdanubian region of Hungary. Attitudes towards main supply chain channels, perceived benefits, and disadvantages influencing online medication purchases were evaluated.

## Methods

In our cross-sectional explorative study, a personally administered survey was used. The characteristics and background of the respondents were measured through the following independent variables: (1) gender, (2) age, (3) level of education, (4) place of residence, (5) average income, (6) internet usage, (7) online purchase habits in general, and (8) self-reported health status.

A Hungarian language survey was developed by the authors (AF and RGV [pharmacists], and MK [psychologist]) in support of this study, based on previous research [38] and a prior pilot study. In the online pilot study, open questions were used, covering the topics of the study survey to map the general attitudes of the prospective sample. In the main study, data were collected directly from Hungarian citizens using the outpatient health services for chronic or acute conditions, between January and March 2018. Participants were considered eligible if they were 16 years of age or older, and were excluded if they were unwilling to participate in the survey.

Trained research associates administered the 28-item survey. It consisted of an introductory paragraph on the aims of the survey, information on confidentiality, and anonymity followed by 5 main sections: (1) evaluation of channels available for procuring medications, (2) online medicine purchase experiences and attitude, (3) internet use, (4) health status and medication use, and (5) demographics. The study protocol was approved by the institutional review board (approval number 6835). In the survey, 5-point Likert-type scales, multiple-response, and multiple-choice questions were used. The English translated version of the survey (Multimedia Appendix 1) and the original Hungarian version (Multimedia Appendix 2) are provided as supplementary material. Statistical analyses were conducted using the SPSS software version 22. Descriptive statistics was used to describe respondent characteristics.

## Results

### Respondent Characteristics

Trained research associates approached a total of 1366 patients. This resulted in the completion of 1055 surveys indicating a response rate of 77.23% (see respondent characteristics in Table 1). The distribution of female (539/1055, 51.09%) and male (516/1055, 48.91%) respondents was nearly equal. Our sample consisted of people obtaining outpatient health service for chronic or acute conditions, thus it represents patients rather than the general population regarding age and number of medications used. The mean age was 45 (SD 17.36) years. Nearly half (456/1055, 43.22%) of the respondents reported to have chronic health conditions and a majority reportedly used at least one medication regularly.

Our survey sample represents the Hungarian society regarding the level of education [39]. According to recent statistics, 72% of individuals in the European Union member states accessed the internet daily, while 57% of Europeans (aged 16 to 74 years) ordered or bought goods or services over the internet for private use. Accordingly, our sample represents the European population for internet use and online purchases [40].

**Table 1:** Respondent demographic, health status, and internet use characteristics (N=1055).

Variable		Value
Age (years), mean (SD)^a^		45.08 (17.36)
**Gender, n (%)**		
	Female	539 (51.09)
	Male	516 (48.91)
**Education, n (%)**		
	Completed primary school	68 (6.45)
	Graduated high school	656 (61.18)
	Graduated college or university	329 (31.18)
	Advanced (PhD, Doctor of Liberal Arts)	2 (0.19)
Patients with chronic conditions, n (%)		456 (43.22)
Number of regular medications per patient, mean (SD)		1.55 (2.63)
Number of regular medications per patient, range		0-25
**Frequency of internet use, n (%)**		
	Daily	737 (69.86)
	Weekly	150 (14.22)
	Never	168 (15.92)
**Frequency of online shopping, n (%)**		
	Regularly	203 (19.24)
	A few times	515 (48.82)
	Never	337 (31.94)

^a^Median 45 years, range 16-89 years.

### Evaluation of Supply Chain Retail Channels, Previous, and Prospective Purchases

Attitudes towards the 3 main supply chain retail participants: conventional community pharmacy units, nonpharmacy units, and internet pharmacies were evaluated. The respondents were asked to rate pharmacies, nonpharmacy units (eg, petrol stations) and internet sources on a 5-point Likert scale.

They were asked to express, according to their opinion, how appropriate were these sources regarding the purchase of medication. A score of 1 was given for “not appropriate at all” and 5 for “entirely appropriate.” The results are shown in Table 2. A repeated measures analysis of variance (ANOVA) was conducted on the sample, and significant differences were found. The respondents accepted retail pharmacy units as the most appropriate source of medications, while they exhibited neutral attitudes toward nonpharmacy units and rejected internet pharmacies (*F*_1.95,2056.66_=1776.78, *P*<.001)

According to our results, 872 (82.65%) of the respondents were aware that medications could be obtained online, while only 44 (4.17%) used the internet for the purchase of medication at least once. However, this number is likely to increase in the future as numerous patients were open to prospective online purchases: 100 (9.47%) stated they were very likely, and 146 (13.83%) noted that they were likely to purchase medications online in the future.

### Perceived Benefits and Disadvantages

Based on the results of the pilot study, 9 statements regarding the potential benefits and disadvantages were measured to determine the factors influencing the attitudes toward online medication purchase. The respondents were asked to evaluate each statement (Table 3) on potential benefits and disadvantages regarding their own attitudes on a 5-point Likert scale. A score of 1 was given for “I don’t agree” and 5 for “I agree.”

**Table 2:** A summary of attitudes towards the 3 main supply chain retail channels using a 5-point Likert scale. A score of 1 was given for “not appropriate at all” while 5 was given for “entirely appropriate.”

Retail channels	Mean (SD)
Pharmacy	4.79 (0.53)
Nonpharmacy units	2.94 (1.38)
Internet	2.25 (1.42)

**Table 3:** A comparative evaluation of potential benefits and disadvantages of online drug shopping.

Parameters		Evaluation, mean (SD)
**Potential benefits**		
	Convenient	4.29 (1.07)
	People who cannot get to a pharmacy can also purchase products	4.18 (1.11)
	I can purchase medicines after opening hours	4.1 (1.19)
	I can access products which are otherwise not available for me	3.34 (1.43)
	Fast	3.71 (1.29)
	Products can be compared faster and more easily than in the pharmacy	3.15 (1.34)
	Inexpensive	2.87 (1.21)
	I can get more information compared to the pharmacy	2.85 (1.43)
	I can get products with better quality compared to the pharmacy	2.23 (1.19)
**Potential disadvantages**		
	It is easier to abuse preparations	4.24 (1.13)
	There is no control, so I can get products that I do not need or worsen my condition	4.22 (1.06)
	I do not get proper information regarding the use of the products	3.86 (1.06)
	Due to the delivery time, I'm getting the drug later compared to a pharmacy	3.80 (1.15)
	The source of the product is not reliable	3.78 (1.27)
	It is hard for me to choose between the great numbers of products	3.70 (1.25)
	I do not get the right product	3.65 (1.31)
	I receive counterfeit medicine	3.61 (1.25)
	The quality of the product is lower compared than in local pharmacies	3.20 (1.29)

**Table 4:** The results of a correlation analysis between demographic factors, internet usage behavior, and prospective online drug purchase attitude.

Parameter	Prospective online medication purchase attitude
Age	–0.28
Average time spent on the internet	0.31
Internet purchase frequency in general	0.37
Settlement size	0.07
Level of education	0.20
Average income	0.06

The reliability of the answers on the benefits and disadvantages were calculated, and Cronbach’s alpha was determined (benefits alpha=.76, disadvantages alpha=.84) suggesting the reliability values are satisfying. The reactionary attitude regarding the online purchase of medicine was weighted, and a relative attitude rate was computed with a mean of –0.37 (SD 1.25). There was a weak but still significant tendency toward rejection (*t*_1054_=9.64, *P*<.001). Our results showed that there were several factors positively influencing the respondents’ attitude toward online medication purchase, but they still tended to reject this source of drug acquisition. Linear regression analysis was conducted to measure the predictive power of the reported attitudes regarding the willingness to purchase medication online. These results show that attitudes have significant predictive power (*F*_2,1054_=224.87, *P*<.001, R^2^=.299).

### Attitudes Towards Prospective Online Purchases

Our study could not substantiate a clear association with purchasing medications online, due to the small ratio of respondents with previous purchasing experience. However, we did evaluate the willingness to purchase medications on the internet. The correspondence of demographic factors, internet usage behavior, and prospective online drug purchase attitude was examined (see Table 4). A correlation analysis indicated that a significant correlation was found between age, average time spent on the internet, internet purchase in general, settlement size, and level of education.

A linear regression analysis was conducted using a stepwise method to measure the effect of correlating factors on willingness to purchase medications online. According to our results, the factor of internet purchase in general, level of education, and age together, will predict the attitude toward the online purchase of medications (*F*_3,1053_=65.83, *P*<.001, R^2^=.158). A further linear regression analysis was conducted to determine the predicting factors of online purchase behavior, in general. Our results showed the time spent on the internet, age, and education determines the general online purchase (*F*_3,1053_=292.36, *P*<.001, R^2^=.455). Also, the time spent on the internet is highly determined by age and the level of education (*F*_2,1053_=445.13, *P*<.001, R^2^=.459). Based on our results, an explanation model was developed. According to our results, the attitude toward online medication purchase can be explained by the factor of general online purchase behavior, which is determined by the time spent on the internet, which has a strong correspondence to age. The level of education will predict all the other factors, and through this, have a general impact on online medication purchase attitude.

## Discussion

### Consumers Purchasing Medicines Online

According to several surveys, the percentage of people purchasing medicines online varies as published data differs, due to type of product, sample population, education, and income or substance abuse status [10,12]. Thus, the authors aimed to summarize previously published data and key findings on patients procuring medications and health products on the web and perceptions regarding internet pharmacies.

Table 5 summarizes recent scientific data on online medication purchasing of the general population published between 2012-2017. Accordingly, it does not contain: data presented in the previous systematic review by Orizio et al [10], surveys focusing solely on online and mail-order pharmacy users, prescription drugs’ customers, and patients participating in prescription refill programs. Questionnaires from a small sample size or specific patient groups (eg, drug abusers, people buying illegal drugs, men obtaining phosphodiesterase-5 inhibitors) were excluded. Furthermore, nonpeer reviewed publications and estimates were not included, mostly to eliminate bias. Studies not representing the current web used by the general population (eg, early reports and studies focusing on the dark web) were also excluded. It must be noted, the differentiation of actual product categories purchased online is somewhat difficult, as approved medicines, medicinal products, and dietary supplements are often measured as a single category in articles evaluating consumers purchasing from online pharmacies.

Several authors have studied the number, attitude, and characteristics of online pharmacy users. Although internet pharmacies have been in business for nearly two decades, there is only limited scientific evidence published regarding the prevalence of online pharmacy use by the general population [42]. In countries where the internet pharmacy market is dominated by retail pharmacy chains selling prescription-only medications and offering refill programs (eg, US, United Kingdom, Germany), patient characteristics differ from nations in which only nonprescription products can be marketed (eg, Central European Union) through the internet.

**Table 5:** Summary of recent studies on the prevalence of purchasing drugs and dietary supplements online.

Reference	Location (year of data collection)	N^a^	Sample population	Survey method	Respondents purchasing health products online (%)
Abanmy [41]	Saudi Arabia (2013-2014)	633	Random sample of internet users	Online	2.7
Alfahad et al [42]	Saudi Arabia (2014-2015)	346	Random sample of internet users	Online	1.4
Szekely et al [43]	Romania (2010-2011)	253	Community pharmacy patients	Personally administered	8.3
Desai et al [37]	USA (2007)	5074	Internet users	Data from HINTS^b^ national dataset	14.5
Brown and Lee [44]	USA (2002-2010)	88,240	Noninstitutionalized individuals	Data from MEPS^c^ national dataset	0.5^d^
Fittler et al [38]	Hungary (2010-2011)	422	Hospital patients	Personally administered	8.4
Mazer et al [45]	USA (2007)	1657	Emergency department patients	Personally administered	5.4^e^

^a^N refers to number of respondents.

^b^HINTS: Health Information National Trends Survey.

^c^MEPS: Medical Expenditure Panel Survey.

^d^Refers to prescription medication.

^e^Refers to medication in general.

As noted by numerous authors who have reviewed the published data, it is most difficult to measure the number of online pharmacies and their customers, especially illegitimate ones [10]. Existing research only provides estimates of the scale of the online purchase of medicine [46].

A US survey, in a cohort of 443 online pharmacy users, representing an average of 1.5 million individuals annually, found that, compared with nonusers, online users were older, are more likely to have private insurance, had more prescriptions, possessed a higher family income, and were more educated [44]. Atkinson et al [47] found that age and marital status was associated with online buying. In 2007, Desai and colleagues [37] documented that about 14.5% of the US population used the internet to obtain medications or vitamins, and these individuals were more often married, white, 50-64 years old with varying levels of college education and an income of US $75,000, or more. It should be noted, mail-order pharmacies are required by certain health plans in the US, which may result in a higher number of patients using online mail pharmacies for maintenance medications [48], compared to other countries. A Hungarian survey of hospital patients has shown in which 8.4% of the respondents ordered drugs or dietary supplements online and 3.7% of the respondents are considering this option in the future. Gender, age, and educational profile did not significantly affect the experience in ordering health-related products from the internet [38]. An Italian study involving more than 100 adult subjects investigated the use of the internet regarding the search for information on medicines, dietary supplements, and disease. Although 68.5% of the respondents were aware of the possibility to purchase medicines on the internet, only 9.2% expressed a favorable opinion. Interestingly, the number of participants with actual online medication purchase experience was not measured [49]. Mazer et al [45] found, among emergency department patients, 57% reported awareness of online pharmacies and 5.4% used the internet to order medications. Multiple medications and prescription plan significantly influenced online pharmacy use, while no difference in age or student status was observed between users and nonusers. A survey of Saudi citizens showed the online purchase of medicines is not yet widespread, as 23.1% of the respondents were aware of the existence of internet pharmacies and only 2.7% had purchased medicines online. However, the level of satisfaction was high among those who had such experience, and a suitable number of respondents (42.7%) indicated they are willing to try an online pharmacy in the future [41]. Similar results were reported by Alfahad et al [42] throughout the kingdom, as a clear majority of the Saudi respondents have not yet heard about online pharmacies (82.6%), and very few (1.4%) have purchased medicinal products online, however about two thirds (66.4%) were enthusiastic to utilize the online options in the purchasing of medicines. On the other hand, a Romanian survey found only a minority (3.2%) of the respondents have not heard of the possibility of purchasing medicines online, 8.3% have already purchased, moreover 7.1% intended to do so in the future [43].

A systematic review by Orizio et al [10] investigated the available evidence regarding online pharmacies, published between 2003-2010. The authors summarized population surveys on consumers perceptions and attitudes yet could not find consistent information regarding the number of consumers and their characteristics [10]. According to another review by Orsolini et al [50] a range of variables must be considered in profiling online pharmacy customers. Most online customers were reported to be young, Caucasian, and individuals without any health insurance. However, there are variations in gender and age depending on the type of medication purchased. Women and more educated individuals were associated with the online search of health-related information, and, conversely, subjects with a low literacy level are prone to purchase from illegal websites.

The above studies provide important clues into what genres of patients and consumers may be more likely to purchase products from online pharmacies. At the same time, we must admit, it is somewhat difficult to profile the “typical online pharmacy customer,” because users are as diverse as the medications they are looking for [12]. This study has identified a knowledge deficit and lack of scientific evidence regarding studies on patients’ attitudes towards internet pharmacies. Furthermore, we believe the additional scientific evidence is necessary to plan, implement, and evaluate prevention campaigns aiming to facilitate the safe use while navigating the online pharmaceutical market.

### Principal Results

Patients’ attitude towards online pharmacies and purchasing health products on the internet is a key element of maintaining the integrity of the medication supply chain and protecting patients from digital iatrogenesis [51]. Policy makers and authorities must be aware of how medications are different from most items purchased on the internet, as they directly have an impact on one’s health [37].

Users of online pharmacies, whether legitimate sites or not, are purchasing medications used for both acute and chronic conditions, including medications of abuse. Without the appropriate advice and the supervision of the medical doctor or the pharmacist, all drugs (prescription only and over-the-counter) and even dietary-supplements can cause harm. The primary concerns are insufficient or incorrect information about the patient’s health status and medications, inappropriate self-diagnosis, or incomplete management of drug-related issues, such as polypharmacy, therapeutic duplications, adverse events, and drug-to-drug or drug-herbal interactions [45]. Obviously, illegitimate online pharmacies pose a definite threat to patients through the marketing of counterfeit medications and substandard products. However, even legitimate actors possess issues associated with their use [37].

Given the variable quality of internet pharmacies, it is critical to identify patients’ vulnerability and develop targeted campaigns to educate the public properly. Our results may support the development of patient-centered interventions by identifying consumer characteristics associated with willingness towards purchasing medications online.

A majority (872/1055, 82.65%) of the respondents were aware medications can be obtained online. In our study sample, 44 (4.17%) reportedly used the internet for purchasing medications previously, and this number is likely to increase in the future, as numerous patients were more likely to purchase medications online in the future (23.3%). Attitudes towards the three main supply chain retail channels showed respondents accepted community pharmacy units as the most appropriate source of medications, yet showed neutral attitudes toward nonpharmacy units and rejected internet pharmacies. The comparative evaluation of potential benefits and disadvantages of online drug shopping demonstrates, there are several factors positively influencing the respondents’ attitude toward online medication purchase (eg, convenience, about individuals who cannot get to a pharmacy can purchase products, possibility to purchase medicines after opening hours). Interestingly, they still tend to reject this source of drug acquisition. Seemingly, Hungarian patients are not yet open towards online medication purchase, however, based on our findings, attitudes will likely change as more individuals gain experience in buying products or services online.

Based on our results, the main factors influencing the willingness towards online medication purchase was determined. We found, internet usage and online purchase behavior, in general, will predict the attitude toward online medication purchase. Essentially, this means the respondents who use the internet and purchase goods online will be more likely to purchase medications online. These 2 factors are highly influenced by age, meaning the younger generation is much more involved in the online market. In addition, the level of education and attitude will determine the medication purchase behavior. People possessing higher level college degrees and having a more positive attitude regarding online medication purchase, are more likely to do so. The attitude ratings are also related to age, mostly in aspects of benefits (*r*=.23). Based on these data, we summarize how online medication purchase is not an isolated phenomenon, but highly integrated with the behavioral tendency in which individuals often try to manage increasingly things online. Given this a general tendency throughout society, we believe efforts against online medication purchase, in general, is pointless. However, it is highly essential to develop a stronger control over online pharmacies and provide education regarding the responsible internet purchase behavior. Previous studies have noted that we must further emphasize the opportunity in support of health providers (general practitioners, pharmacists, nurses) to help patients navigate potential internet purchases, in the prevention of medication incidents generated using unapproved and illegitimate online pharmacies [37,38].

### Strengths and Limitations

In this study, the potential misunderstanding of the survey was eliminated by personally administering the survey and the use of trained research associates. Compared to numerous previous studies, our method makes it possible to identify 2 seemingly similar, but different product categories (medications and dietary supplements) separately. In the beginning of the survey, the special attributes of medications were measured and discussed for future reference. In our sample, we gathered results from a balanced respondent population, representing the patient population regarding age, gender, and education. Previous survey results regarding online consumers may not reflect the prevalence and the attitude of the general population because internet use is strongly related to several demographic variables. Accordingly, by using personally administered surveys, our study eliminates such bias.

This study does have some limitations. Online pharmacy use was self-reported, therefore, subject to recall bias and untruthful reporting by the individual, which may underestimate actual prevalence. Legitimate and illegitimate actors are not differentiated in our study, mainly, since online sellers may mislead customers or not be able to differentiate between them [12,52]. Our study was performed in hospitals, general practitioners’ offices, and community pharmacies throughout Southern Hungary. Consequently, it represents the national patient population but not the entire Hungarian population. However, this can be a potential strength, as patients are more likely to purchase medications and they are also vulnerable to the potential dangers associated with internet pharmacies. Prevalence and attitudes of inpatients were measured and published previously by the authors [38].

### Conclusions

Our results support our hypothesis: the use of the internet to purchase medications is present and national results are in correlation with international data. Despite a weak, but still significant tendency towards rejection to online pharmacies was identified, a reasonable number of patients were planning to purchase medications on the internet in the future.

We aimed to identify drivers in support of the purchase of online medication to develop targeted campaigns informing patients vulnerable to illegal websites. Based on the literature review and our study results, it is difficult to profile consumers turning to the internet pharmacy market because users are likely just as diverse as the treatments they are looking for. However, following the evaluation of prospective online drug purchase attitude, we arrived to the conclusion that respondents who use the internet more frequently to shop online will be more likely to purchase medications on the internet. Furthermore, age and education determine in the medication purchase behavior. Our results ideally will soon support educational interventions promoting safe online medication procuring practices and provide valuable data regarding patients’ attitudes.

It is highly recommended that the participants of the health care system, both throughout the institutional and outpatient settings, begin documenting procurement information during the medical examination, interview, and anamnesis of their patients. This will ensure that more robust and informative data will be made available regarding the effective penetration of this relatively novel distribution channel of the drug supply system.

Future research should focus on exploring adverse effects resulting from medications purchased online. New emerging technologies, such as machine learning algorithms applied to “Big Data,” is the basis for a new area of research referred to as, “digital” surveillance or “infoveillance” [53]. Accordingly, the actual patient safety risk in outpatients can be identified (1) within the health care system by collecting data obtained during medical examinations and anamnesis, (2) by the evaluation of patient records, or (3) performed online by novel data-science methods.

Improved patient-provider communication, promotion, and education campaigns are needed to inform and educate the public on the safe use of internet pharmacies. These initiatives can prevent threats to patient safety. Targeted interventions by pharmacists (medication review or reconciliation, professional advice regarding the evaluation of online distributors, and differentiate legal and illegal medication suppliers) are potential prevention strategies which must be emphasized, including the effective implementation in everyday practice [54].

## Appendix

Multimedia Appendix 1 Survey in English.

Multimedia Appendix 2 Survey in Hungarian.

## Abbreviations

ANOVA: analysis of variance
HINTS: Health Information National Trends Survey
MEPS: Medical Expenditure Panel Survey
