# Supplementary material for: Consumers Turning to the Internet Pharmacy Market: Cross-Sectional Study on the Frequency and Attitudes of Hungarian Patients Purchasing Medications Online
Source: J Med Internet Res. 2018 Aug 22;20(8):e11115. doi: 10.2196/11115 (PMC6125612; doi:10.2196/11115)
Supplement: Multimedia Appendix 1 [file jmir_v20i8e11115_app1.pdf]

Questionnaire regarding frequency and attitudes of patients purchasing medications online

(28 item survey)

The survey was developed by the instructors of the Department of Pharmaceutics and Central Clinical Pharmacy, Faculty of Pharmacy, University of Pécs and instructor from the Institute of Psychology, Faculty of Humanities, University of Pécs to evaluate patient and consumer safety. Our aim is to measure the experience and attitude of outpatients regarding the online sale of medical products and internet pharmacies.

The survey is anonymous and the participation is voluntary and has no consequences for the patients.

The results of the survey will be used to further evaluation of the identified and high risk internet pharmacies and products and for scientific purposes.

The data collected during the research will be handled according to the rules of scientific research ethics and none of the participants can be identified from the results and publications (it will not contain any data regarding name, birth date, etc.).

Study protocol was approved by the University of Pécs Clinical Centre Regional Committee for the Research Ethics on the 8th of September, 2017 (approval number: 6835).

If you have further questions regarding the survey or the scientific research topic, please contact the following leaders of the research:

- Dr. András Fittler, Department of Pharmaceutics and Central Clinical Pharmacy, Faculty of Pharmacy, University of Pécs Address: 7624 Pécs, Honvéd st. 3., Telephone number: +36 20 5566 509, or 72/536 284, e-mail: fittler.andras@pte.hu
- Dr. Mátyás Káplár, Institute of Psychology, Faculty of Humanities, University of Pécs Address: 7624 Pécs, Ifjúság st. 6., Telephone number: +36 20 8280113, e-mail: kaplar.matyas@pte.hu

Completion of the survey takes about 10-15 minutes.

I. Evaluation of channels available for procuring medications

1. Did you know that medicines are products ...

	Yes	No
1. with high quality which was evaluated before getting to market by a pharmaceutical authority.		
2. with efficacy proven with clinical trials.		
3. with strictly controlled manufacturing and distribution.		
4. that their path can be traced from the manufacturer to the patient.		
5. that their ordering and dispensing is under the supervision of health care professionals.		

2. How appropriate would you rate the following options regarding the purchase of medicines?

(use a 1 to 5 point Likert scale, where 1 “not appropriate at all”, 5 “totally appropriate”)

- Pharmacy
- Herbal shops, petrol station
- Internet

3. Have you heard that medicines can also be purchased online?

- Yes
- No

II. Online medicine purchase experiences and attitude

1. Evaluate the potential advantages of online drug shopping:

(use a 1 to 5 point Likert scale, where 1 “I don’t agree”, 5 “I agree”)

- Fast
- Convenient
- Inexpensive
- Products can be compared faster and more easily than in the pharmacy
- I can get more information compared to the pharmacy
- People who can’t get to a pharmacy can also purchase products
- I can purchase medicines after opening hours
- I can access products which are otherwise not available for me
- I can get products with better quality compared to the pharmacy

2. Evaluate the potential disadvantages of online drug shopping:

(use a 1 to 5 point Likert scale, where 1 I don’t agree, 5 I agree)

- I don’t get the right product
- I do not get proper information regarding the use of the products
- The source of the product is not reliable
- It is easier to abuse preparations
- Due to the delivery time, I'm getting the drug later compared to a pharmacy
- There is no control, so I can get products that I don’t need or worsen my condition
- It is hard for me to choose between the great numbers of products
- The quality of the product is lower compared than in local pharmacies
- I receive counterfeit medicine

3. How likely will you buy medicines over the internet in the future?

(use a 1 to 5 point Likert scale, where 1 “Unlikely”, 5 “Very likely”)

4. How great is the size of the problem caused by counterfeit medicines in Hungary today?

(use a 1 to 5 point Likert scale, where 1 “Not a problem”, 5 “It is a big problem”)

III. Internet use

1. How much time do you spend online on the Internet?

- I do not use Internet
- 1-2 hours a week
- 1-2 hours a day
- More hours a day
- Other: …….

2. Do you use Internet for purchases? (in general, regardless of product or service)

- No
- I have used once or twice
- I have used several times
- I use it frequently
- Other: …….

IV. Health status and medication use

1. How would you rate your health status over the past 1 year?

(use a 1 to 5 point Likert scale, where 1 Awful, 5 Excellent)

2. Do you have a chronic disease?

Yes

No

NA (no answer)

3. How many medications do you take regularly?

Number: ……

4. If you have a chronic condition please tell us what is the disease?

Disease: …………………

5. If you have a chronic condition please tell us how do you purchase the medicine you are taking for the disease?

Anatomical Therapeutic Chemical Classification System (WHO - ATC main groups)		I do not have a disease belonging to that group	Purchased from “brick and mortar” pharmacy	Purchased outside of a pharmacy	Purchased from the Internet
A	Alimentary tract and metabolism				
B	Blood and blood forming organs				
C	Cardiovascular system				
D	Dermatologicals				
G	Genito-urinary system and sex hormones				
H	Systemic hormonal preparations, excluding sex hormones and insulins				
J	Antiinfectives for systemic use				
L	Antineoplastic and immunomodulating agents				
M	Musculo-skeletal system				
N	Nervous system				
R	Respiratory system				
S	Sensory organs				

6. How typical is of you to get information about your disease from the internet?

(use a 1 to 5 point Likert scale, where 1 not typical, 5 very typical)

7. How likely do you think that you would self-medicate yourself based upon the information found on the internet?

(use a 1 to 5 point Likert scale, where 1 not typical, 5 very typical)

8. Do you take medication in case of acute disorders? (e.g.: common cold, headache)

- Yes
- No
- NA (no answer)

9. If yes, where do you purchase your medication?

- Pharmacy
- Herbal shops, petrol station
- Internet

10. How many timed have you used the Internet to purchase medicine?

- Never
- I have used once or twice
- I have used several times
- I use it frequently

11. If you have purchased medicine, what was it and from which site have you used?

Website: ...

12. How many timed have you used the internet to purchase other medical products (e.g.: dietary supplements)?

- Never
- I have used once or twice
- I have used several times
- I use it frequently

13. If you have purchased other medical products (e.g.: dietary supplements), what was it and from which site have you used?

Website: ...

<u>V. Demographics</u>

1. Gender

- Male
- Female

2. Birth date (year)

3. Highest level of education

- Primary school completed
- High school graduation
- College or University graduation

- College+ (PhD – Doctor of Philosophy, DLA - Doctor of Liberal Arts)

4. Settlement hierarchy based upon population

- City with county rights
- Town
- Village

5. Average monthly income

- Less than 250 EURO
- 250 EUR – 500 EURO
- 500 EUR – 800 EURO
- Above 800 EURO
- NA (no answer)

6. Zip code

…

Comments, comments on the survey or topic

(optional)
