# Supplementary material for: Consumers Turning to the Internet Pharmacy Market: Cross-Sectional Study on the Frequency and Attitudes of Hungarian Patients Purchasing Medications Online
Source: J Med Internet Res. 2018 Aug 22;20(8):e11115. doi: 10.2196/11115 (PMC6125612; doi:10.2196/11115)
Supplement: Multimedia Appendix 2 [file jmir_v20i8e11115_app2.pdf]

**Online gyógyszervásárlás gyakoriságát és vásárlási szokásokat vizsgáló kérdőív kérdései**

*(28 kérdésből álló kérdőív)*

Az alábbi kérdőívet a Pécsi Tudományegyetem Gyógyszerésztudományi Kar Gyógyszerészeti Intézetének gyógyszerész oktató és Pécsi Tudományegyetem Bölcsészettudományi Kar Pszichológia Intézet oktató munkatársai állították össze a beteg és fogyasztói biztonság megismerése céljából.

Kérdőíves megkérdezésünk során arra keressük a választ, hogy az egészségügyi szolgáltatást igénybe vevő személyek milyen véleménnyel és tapasztalatokkal rendelkeznek a különböző gyógyszerforgalmazási lehetőségekről.

A válaszadás önkéntes és semmilyen következménnyel nem jár a kitöltőre nézve. Az így nyert adatokat az esetleges veszélyt jelentő internetes gyógyszertárak vagy termékek további vizsgálatára, illetve ezekkel kapcsolatos tudományos kutatási célú elemzések készítésére használjuk fel. A vizsgálat során gyűjtött adatokat szigorúan bizalmasan kezeljük. A vizsgálat eredményei kizárólag olyan formában kerülnek közlésre, amely a résztvevők személyének azonosítására alkalmas személyes adatokat (név, születési dátum stb.) nem tartalmaz.

A PTE-Klinikai Központ Regionális és Intézményi Kutatás-etikai Bizottság a vizsgálatot a 6835. ügyirat-számú döntése alapján 2017.09.08. napján engedélyezte.

Ha Önnek kérdése van a vizsgálattal kapcsolatban vagy további információkat szeretne kapni, akkor az alábbi, vizsgálatért felelős személyekkel veheti fel a kapcsolatot:

- Dr. Fittler András, PTE GYTK Gyógyszerészeti Intézet, egyetemi docens, 7624 Pécs, Honvéd u. 3., telefon: +36 20 5566 509, vagy 72/536 284, e-mail: fittler.andras@pte.hu
- Dr. Káplár Mátyás, PTE BTK Pszichológia Intézet, egyetemi adjunktus, 7624 Pécs, Ifjúság útja 6., telefon: +36 20 8280113, e-mail: kaplar.matyas@pte.hu

A kitöltés ideje 10-15 perc.

**<u>I. Gyógyszer-beszerzéssel kapcsolatos kérdések</u>**

**1. Tudta-e hogy a gyógyszerek...**

| | Igen | Nem |
|---|---|---|
| 1. olyan kiemelkedő minőségű készítmények, melyek biztonságosságát a gyógyszerészeti hatóság előzetesen értékeli. | | |
| 2. hatását klinikai vizsgálatokkal igazolták. | | |
| 3. gyártása és forgalomba kerülésük szigorúan szabályozott. | | |
| 4. útja a gyártótól a betegig pontosan végigkövethető. | | |
| 5. megfelelő rendelését és kiadását egészségügyi szakemberek felügyelik. | | |

**2. Mennyire tartja megfelelő beszerzési formának az alábbi lehetőségeket gyógyszervásárlás tekintetében?**

(azalábbi 1-től 5-ig terjedő Likert skálán, ahol 1 az „egyáltalán nem megfelelő”-t, míg az 5 „Teljes mértékben megfelelő”-t jelöli)

- Gyógyszertár
- Drogéria, benzinkút, szaküzlet (gyógynövény, fitnesz, stb.)
- Internet

**3. Hallott-e már arról, hogy gyógyszereket az interneten is be lehet szerezni?**

- Igen
- Nem

## II. Internetes gyógyszer-vásárlással kapcsolatos vélekedés

**1. Értékelje az internetes gyógyszervásárlás lehetséges előnyeit!:**

(azalábbi 1-től 5-ig terjedő Likert skálán, ahol 1 az „Egyáltalán nem értek egyet”-t, míg az 5 „Teljes mértékben egyetértek”-t jelöli)

- Gyors
- Kényelmes
- Olcsó
- A termékek gyorsabban és egyszerűbben összehasonlíthatóak, mint a gyógyszertárban
- Több információt kapok a termékről, mint a gyógyszertárban
- Olyanok is hozzájuthatnak a gyógyszerekhez, akik nem tudnak eljutni a patikába
- Nyitvatartási időn túl is rendelhetek gyógyszert
- Olyan termékekhez is hozzájuthatok, melyeket amúgy nem kaphatok meg
- A hazai gyógyszertárakban elérhető készítményeknél jobb minőségű terméket kapok

**2. Értékelje az internetes gyógyszervásárlás lehetséges hátrányait!:**

(azalábbi 1-től 5-ig terjedő Likert skálán, ahol 1 az „Egyáltalán nem értek egyet”-t, míg az 5 „Teljes mértékben egyetértek”-t jelöli)

- Nem a megfelelő készítményt kapom
- Nem kapok megfelelő szakmai tájékoztatást a termékek használatáról
- Nem megbízható, nem ellenőrzött forrásból kapom a készítményt
- Könnyebb visszaélni a készítményekkel
- A kiszállítási idő miatt lassabban jutok hozzá a gyógyszerhez, mint ha patikában vásárolnám
- Nincs kontroll, ezért olyan készítményhez is hozzáférhetek, amire nincs szükségem, vagy ronthatja az állapotomat
- A túl sok lehetőségből nehéz a számomra legmegfelelőbbet kiválasztani
- A hazai gyógyszertárakban elérhető készítményeknél rosszabb minőségű terméket kapok
- Hamis gyógyszert kapok

**3. Mennyire tartja elképzelhetőnek, hogy a jövőben az interneten vásároljon gyógyszert?**

(azalábbi 1-től 5-ig terjedő Likert skálán, ahol 1 az „Egyáltalán nem valószínű”-t, míg az 5 „Nagyon valószínű”-t jelöli)

**4. Ön szerint mekkora problémát jelent napjainkban a gyógyszerhamisítás Magyarországon?**

(azalábbi 1-től 5-ig terjedő Likert skálán, ahol 1 az „Nem jelent problémát”-t, míg az 5 „Nagy problémát jelent”-t jelöli)

**<u>III. Internethasználattal kapcsolatos szokások</u>**

**1. Ön mennyi időt tölt átlagosan az interneten?**

- Nem használok internetet
- Heti 1-2 óra
- Napi 1-2 óra
- Napi több óra
- Egyéb: …….

**2. Használja-e az internetet vásárlásra? (Termék és szolgáltatás típustól függetlenül.)**

- Nem
- Vásároltam már 1-2 alkalommal
- Több alkalommal vásároltam már
- Rendszeresen vásárolok az interneten
- Egyéb: …….

**<u>IV. Egészségügyi állapottal és gyógyszereléssel kapcsolatos kérdések</u>**

**1. Hogyan értékelné a saját egészségügyi állapotát az elmúlt 1 évben?**

(azalábbi 1-től 5-ig terjedő Likert skálán, ahol 1 az „Nagyon rossz”-t, míg az 5 „Nagyon jó”-t jelöli)

**2. Van-e krónikus betegsége?**

- Van
- Nincs
- Nem válaszol

**3. Hány gyógyszert szed rendszeresen?**

Szám: ……

**4. Amennyiben van krónikus megbetegedése, kérjük nevezze meg, illetve jelölje, hogy honnan szerzi be a szedett gyógyszereket?**

Megbetegedések: ………………….

**5. Amennyiben van krónikus megbetegedése, kérjük nevezze meg, illetve jelölje, hogy honnan szerzi be a szedett gyógyszereket?**

| ATC hatóanyag csoportok | | Nincs ilyen megbetegedésem, vagy nem szedek | Gyógyszer-tárból | Gyógyszer-táron kívül üzlet, egyéb | Internet |
|---|---|---|---|---|---|
| A | Tápcsatorna és anyagcsere | | | | |
| B | Vér és vérképző szervek betegségei | | | | |
| C | Szív-és érrendszeri betegségek | | | | |
| D | Bőrgyógyászati megbetegedések | | | | |
| G | Húgyúti és ivarszervi, nőgyógyászati betegségek | | | | |
| H | Hormonális zavarok | | | | |
| J | Fertőzéses megbetegedések | | | | |
| L | Daganatos megbetegedések | | | | |
| M | Mozgásszervrendszer megbetegedései | | | | |
| N | Központi idegrendszer megbetegedései | | | | |
| R | Légzőszervrendszeri megbetegedések | | | | |
| S | Érzékszervek megbetegedései | | | | |

**6. Mennyire jellemző önre, hogy megbetegedés esetén interneten tájékozódik a betegségéről?**

(azalábbi 1-től 5-ig terjedő Likert skálán, ahol 1 az „Egyáltalán nem jellemző”-t, míg az 5 „Nagyon jellemző”-t jelöli)

**7. Mennyire tartja valószínűnek, hogy az online megszerzett információk alapján önállóan alkalmazzon gyógyszeres kezelést?**

(azalábbi 1-től 5-ig terjedő Likert skálán, ahol 1 az „Egyáltalán nem jellemző”-t, míg az 5 „Nagyon jellemző”-t jelöli)

**8. AKUT betegségek esetén (pl. megfázás, fejfájás, stb.) szokott-e gyógyszert beszedni?**

- Igen
- Nem
- Nem válaszolt

**9. Ha igen, honnan szerzi be az AKUT problémákra vásárolt gyógyszerét??**

- Gyógyszertár
- Drogéria, benzinkút, szaküzlet
- Internet

**10. Hányszor használta már az internetet GYÓGYSZER-vásárlásra?**

- Soha
- Vásároltam már 1-2 alkalommal
- Több alkalommal vásároltam már
- Rendszeresen vásárolok az interneten

**11. Ha már vásárolt GYÓGYSZERT, mit és milyen honlapról szerzett be?**

Honlap megnevezése: …

**12. Hányszor használta már az internetet EGYÉB EGÉSZSÉGÜGYI TERMÉK vásárlásra (pl.: étrend-kiegészítő)?**

- Soha
- Vásároltam már 1-2 alkalommal
- Több alkalommal vásároltam már
- Rendszeresen vásárolok az interneten

**13. Ha már vásárolt EGYÉB EGÉSZSÉGÜGYI TERMÉKET, mit és milyen honlapról szerzett be?**

Honlap megnevezése:

**V. Demográfiai adatok**

**1. Az ön neme**

- Férfi
- Nő

**2. Az ön születési éve**

**3. Az ön legmagasabb iskolai végzettsége**

- Általános iskola
- Középiskola
- Főiskola / egyetem
- PhD / DLA

**4. Az ön lakóhelye**

- Megyei jogú város
- Város
- Falu

**5. Mennyi az átlagos havi bevétele?**

- 0 – 80 000Ft
- 81 000 - 150 000Ft
- 151 000 - 250 000Ft
- 250 000Ft felett
- Nem válaszol

**6. Az ön irányítószáma**

...

**Megjegyzések, észrevételek a kérdőívvel vagy témával kapcsolatban**

(opcionális)
